# Correction: Combined mechanistic and genetic programming approach to modeling pilot NBR production: influence of feed compositions on rubber Mooney viscosity

**DOI:** 10.1039/d1ra90077c

**Published:** 2021-02-04

**Authors:** Ge He, Tao Luo, Yagu Dang, Li Zhou, Yiyang Dai, Xu Ji

**Affiliations:** School of Chemical Engineering, Sichuan University Chengdu 610065 China jxhhpb@163.com tao.luo@scu.edu.cn +86-028-85405220 +86-028-85405220; Lanzhou Petrochemical of PetroChina Company Limited Lanzhou 730060 China

## Abstract

Correction for ‘Combined mechanistic and genetic programming approach to modeling pilot NBR production: influence of feed compositions on rubber Mooney viscosity’ by Ge He *et al.*, *RSC Adv.*, 2021, **11**, 817–829, DOI: 10.1039/D0RA07257E.

The authors regret that an incorrect version of Fig. 6 was included in the original article. The correct version of Fig. 6 is presented below.

**Fig. 6:**
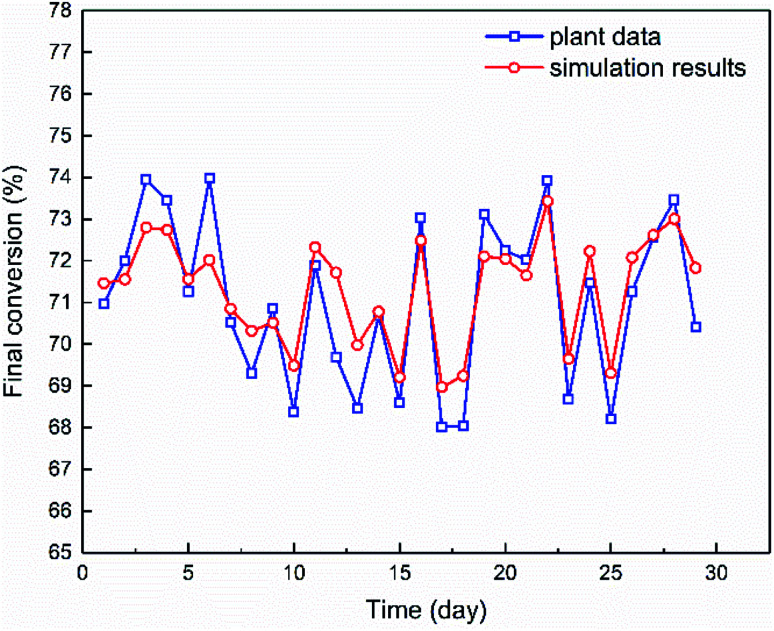
The final conversion of monomers, experimentally measured or simulated *via* the mechanistic model, for the continuous emulsion polymerization process of NBR. The experimental data are measurement results of samples collected periodically over 29 days in sequence.

The Royal Society of Chemistry apologises for these errors and any consequent inconvenience to authors and readers.

## Supplementary Material

